# Hereditary transthyretin amyloidosis: a comprehensive review with a focus on peripheral neuropathy

**DOI:** 10.3389/fneur.2023.1242815

**Published:** 2023-10-05

**Authors:** Loris Poli, Beatrice Labella, Stefano Cotti Piccinelli, Filomena Caria, Barbara Risi, Simona Damioli, Alessandro Padovani, Massimiliano Filosto

**Affiliations:** ^1^Unit of Neurology, Azienda Socio-Sanitaria Territoriale Spedali Civili, Brescia, Italy; ^2^Department of Clinical and Experimental Sciences, University of Brescia, Brescia, Italy; ^3^NeMO-Brescia Clinical Center for Neuromuscular Diseases, Brescia, Italy

**Keywords:** ATTRwt, ATTRv, amyloid, transthyretin, polyneuropathy

## Abstract

Amyloidoses represent a group of diseases characterized by the pathological accumulation in the extracellular area of insoluble misfolded protein material called “amyloid”. The damage to the tissue organization and the direct toxicity of the amyloidogenic substrates induce progressive dysfunctions in the organs involved. They are usually multisystem diseases involving several vital organs, such as the peripheral nerves, heart, kidneys, gastrointestinal tract, liver, skin, and eyes. Transthyretin amyloidosis (ATTR) is related to abnormalities of transthyretin (TTR), a protein that acts as a transporter of thyroxine and retinol and is produced predominantly in the liver. ATTR is classified as hereditary (ATTRv) and wild type (ATTRwt). ATTRv is a severe systemic disease of adults caused by mutations in the *TTR* gene and transmitted in an autosomal dominant manner with incomplete penetrance. Some pathogenic variants in *TTR* are preferentially associated with a neurological phenotype (progressive peripheral sensorimotor polyneuropathy); others are more frequently associated with restrictive heart failure. However, many mutations express a mixed phenotype with neurological and cardiological involvement. ATTRv is now a treatable disease. A timely and definite diagnosis is essential in view of the availability of effective therapies that have revolutionized the management of affected patients. The purpose of this review is to familiarize the clinician with the disease and with the correct diagnostic pathways in order to obtain an early diagnosis and, consequently, the possibility of an adequate treatment.

## Introduction

Although hereditary transthyretin amyloidosis (ATTRv) is globally the most frequent form of familial amyloidosis, it is a rare disorder with a largely variable worldwide prevalence (1–3). It is caused by autosomal dominant mutations in the transthyretin (TTR) gene (*TTR*), located on chromosome 18 (4).

TTR is a protein synthesized in the liver, pancreas, choroid plexus, and retinal pigment epitelium (1, 5). Its primary function is to transport the thyroid hormone thyroxine in the plasma and cerebrospinal fluid and the retinol-binding protein (RBP) bound to retinol in the plasma (1, 5). Pathological dysfunction of TTR protein is related to the aggregation of misfolded proteins causing extracellular deposition of amyloid-insoluble fibrils and local tissue damage (6). While wild-type TTR amyloidosis (ATTRwt) is an acquired condition that mainly affects the heart, develops with age and commonly affects men over the age of 60, ATTRv provides a wide range of clinical presentations that differ for age of onset, organ involvement, and severity of disease (7). It may present with infiltrative cardiomyopathy (hereditary transthyretin amyloidosis cardiomyopathy, or ATTRv-CM) and/or length-dependent sensory motor polyneuropathy (hereditary transthyretin amyloidosis polyneuropathy, or ATTRv-PN), variably associated with autonomic dysfunction (7). However, ATTRv is a multisystem disorder, and clinicians should always investigate possible eye, kidney and gastrointestinal involvement (8–12). The heterogeneity in clinical presentations depends mostly on genotype and ethnicity (13, 14). Thanks to the institution of an online registry for mutations in ATTRv, awareness of genotype-phenotype correlation has been rising in the past decade (13). Regarding global distribution, endemic and non-endemic areas have been described (14–17). Patients from endemic areas (e.g., Portugal, Sweden, Japan) usually present with early-onset involvement of small nerve fibers, while late-onset length-dependent sensory-motor polyneuropathy is frequent in non-endemic areas (14–17). In this review, we discuss the latest acquisitions into the pathology and clinical aspects of ATTRv, with a focus on ATTRv-PN and its management and treatment.

## Epidemiology

The global prevalence of ATTRv is estimated to be around 10,186 people, with a range between 5,000 and 38,000 (2). The so-called endemic clusters are described in Portugal, Sweden, Brazil and Japan where some specific endemic sub-regions (e.g., area of Póvoa do Varzim/Vila do Conde, Skellefteå, and Piteå, Nagano/Kumamoto/Ishikawa) were also reported (2, 17–22). Prevalence in endemic countries ranges from 0.9 to 204 (per 1 000 000 persons) countrywide, yet in endemic sub-regions, it may reach 1,631 (per 1 000 000 persons) (19).

While smaller endemic foci were also reported in Cyprus and Mallorca (15, 16), in the rest of Europe, the incidence of ATTRv is highly variable with countrywide lower rates (2, 19). In Italy, the prevalence is around 4.33/million with regional clusters in Tuscany, Latium and mainly in Southern Italy as Sicily, Calabria, and Puglia (3, 23). However, data from the Italian Registry may be underestimated because of the voluntary participation and the high rate of unrelated patients, suggesting that relatives of affected ones did not always undergo familial segregation (3).

In the last few years, an increasing number of ATTRv late-onset cases have been reported in many countries across Europe, the USA, China, and India (2, 14). With the growing awareness of the disease, the more frequent use of genetic testing, and the benefits of the currently available disease-modifying therapies, the incidence and prevalence of ATTRv are likely to increase over the years, especially in regions where it is not endemic.

The age of onset is usually between the 2nd and 5th decades of life in endemic areas, while late-onset cases (7–8th decade) are more often reported in non-endemic countries (1).

ATTRv affects males and females usually with no significant gender prevalence, yet the parent-of-origin effect in carriers is hypothesized because maternal inheritance of the mutation seems to be related to a higher risk of disease (24). However, in families with late-onset disease, a male predominance has been reported (25).

## From biology to pathophysiology

TTR is an evolutionarily conserved carrier protein synthesized in the liver, choroid plexus, and retinal pigment epithelium (1, 5). It was originally called “prealbumin” because of its pattern of migration at electrophoresis (ahead of albumin) (26). However, this term is no longer used as it may be misleading because TTR has no structural relationship with albumin and doesn't share features with “proalbumin” or “other prealbumins”.

Although its main function is to transport thyroid hormones in the plasma and cerebrospinal fluid and to play a role in vitamin A transport, it is also implicated in many cellular processes such as autophagy, proteolysis, nerve regeneration, and glucose homeostasis (27).

### Genetics

The *TTR* gene is located on chromosome 18 (18q11.2–q12.1) and spans about 7.0 kilobase pairs (26, 27). First sequenced in 1974 by Kanda et al., it consists of four exons and three introns (4, 26, 27). TTR protein structure is a stable homo-tetramer (28, 29). Each subunit contains 124–128 amino acids arranged in eight antiparallel sheets (28, 29). A double-barreled hydrophobic channel forms two binding sites for transport function (28, 29).

More than 150 mutations in the *TTR* gene have been described in literature and online databases such as the Mutations in Hereditary Amyloidosis Database (http://www.amyloidosismutations.com/) (13). Most of them are point mutations, mainly missense pathogenic variants (30). However, a small amount does not lead to an amyloidogenic pathway, and exceptionally few changes (e.g., T119M) have shown a protective role by stabilizing TTR structure and reducing cardiovascular risk (31–33).

**Figure 1:**
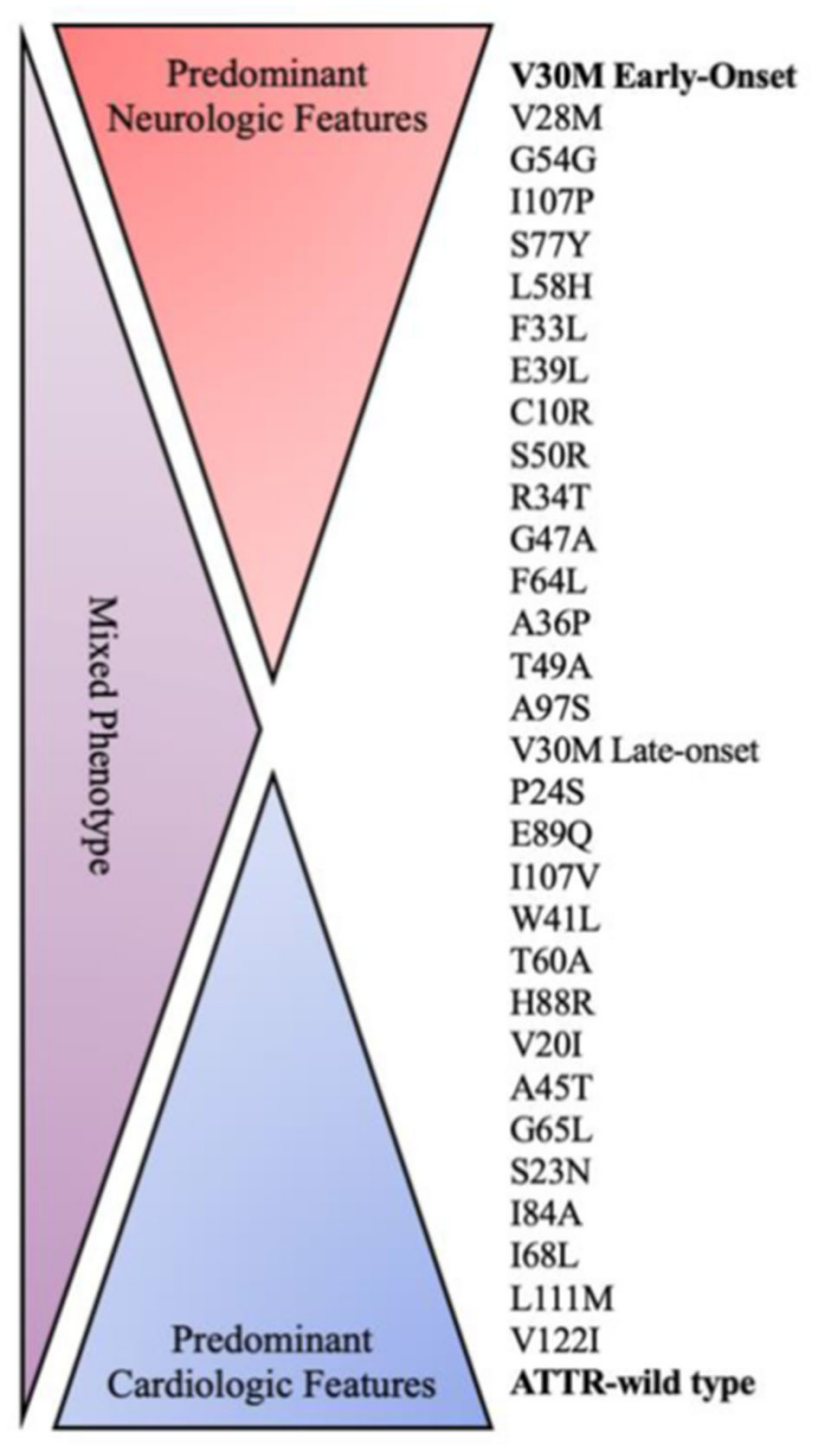
Genotype–phenotype correlation in ATTR amyloidosis. ATTR, amyloid transthyretin; WT, wild type.

Every mutation shows a different pattern of clinical presentation including variable heart and nerve involvement, age of onset, and disease progression (Figure 1). Some pathogenic mutations may be associated with prominent peripheral nerve involvement (ATTRv-PN) as Val30Met, Ala97Ser and Ser50Arg, while others are predominantly linked to cardiomyopathy (ATTRv-CM) such as Ile68Leu, Val122Ile, Thr60Ala and Leu111Met (7, 16, 34). However, most of the known mutations show a mixed phenotype with neurological and cardiological symptoms and signs, with clinical heterogeneity also evident within subjects sharing the same mutation because of the incomplete penetrance (7, 16, 34).

The Val30Met variant, resulting in the substitution of valine by methionine at position 30 on the TTR protein, was the first identified mutation of the *TTR* gene (35). It is the most frequently reported both in endemic and non-endemic areas, being responsible for about 70% of ATTRv (7, 14, 30, 35).

Val30Met phenotype is characterized by familial polyneuropathy associated with autonomic dysfunction (35, 36). In subjects from endemic areas, penetrance is usually high and could be about 90% at 80 years of age (37). Symptom onset occurs usually earlier than in those affected by other mutations, according to the THAOS study (7). However, phenotype expression varies with ethnicity and age. At age 50, penetrance is 60% in Portuguese households but only 18% and 11% in French and Swedish households, respectively (37, 38).

Clinical variability may depend on several genetic and environmental factors, but they are not entirely established, and further studies are needed (37, 38). The hypothesis of a common founder is suggested by haplotype features shared among Spanish, Portuguese, and Japanese patients from Kumamoto and Nagano (39). Penetrance is significantly higher when the pathogenic variant is inherited from the mother, and a sort of anticipation phenomenon (higher penetrance in younger generations) was observed more frequently in descendants of affected women (40). Indeed, patients who inherited the maternal *TTR* mutation manifested earlier symptoms of disease than those with an affected father in the first global observational survey of ATTRv (THAOS–Transthyretin Amyloidosis Outcome Survey) (7). Therefore, a parental imprinting phenomenon and a role for mitochondrial genome in influencing expression of the *TTR*-Val30Met mutation have been hypothized as well as a role for non-coding variants in contributing to *TTR* gene expression and, consequently, phenotype expression (41).

The variant Val122Ile is another widespread pathogenic change more frequently detected in the USA among African American people with a prevalence of 3–4% and in West African populations (7, 30, 42). It mainly results in a predominant cardiac phenotype consisting of restrictive hypertrophic cardiomyopathy with only mild neurological involvement, raising the risk of heart failure in carriers (42, 43).

Some other mutations have been associated with small family groups with common origins, including Thr60Ala in Northern Ireland, Phe64Leu in Sicily, Ser50Arg in Mexico, Ser77Tyr, Ser77Phe in France and Ala97Ser in Taiwan (17–21).

### Pathology and pathophysiology

ATTRv is a protein misfolding disease characterized predominantly by reduced stability of TTR tetramers and increased dissociation into monomers (44). Consequentially, TTR fibrils are predisposed to misfolding, abnormal aggregation, and subsequent extracellular deposition, as happens in other systemic amyloidoses. The accumulation at different sites eventually leads to progressive multiorgan dysfunction (7–12).

The biochemical aspects of the amyloid fibrils may appear different depending on the age of onset and the specific causal mutation (6, 45). The pathological hallmark of an early-onset ATTRv Val30Met mutation is full-length amyloid fibrils (type B) usually long and thick (6, 45). On the other hand, late-onset ATTRv Val30Met forms display a mixture of full-length and TTR non fibrillar fragments (Type A) with short and thin aspect (6, 45, 46). Similarly, other mutations resemble this last type of morphological aspect of amyloid deposits (6, 45, 46). Different types of amyloid fibrils have a different affinity to Congo red staining: type A fibrils display a weaker congophilia leading to a poor possibility in detecting amyloid deposition in the biopsies of late-onset patients (47).

The accumulation of amyloid deposits in both endoneurium and endoneural blood vessels, added to an early disruption of the blood-nerve barrier, is the leading cause of nerve damage (48). The type of nerve fibers involved could vary depending on the age of onset (48). While in the early-onset forms small nerve fibers are primarily damaged, in the late-onset forms large fibers are preferentially involved, with relative sparing of small myelinated and unmyelinated fibers (48).

Moreover, amyloid deposition involves dorsal root ganglia (DRG) and sympathetic ganglia, with different impacts according to the onset of Val30Met-related ATTRv (49). In fact, in the early-onset cases, neuronal cell loss is more severe in sympathetic ganglia than DRGs, vice versa in the late-onset forms (49).

Pathological findings in the Val30Met variants also suggest that the initial site of amyloid deposition may be the DRGs and autonomic ganglia with subsequent spreading along the entire length of the peripheral nerves through the proximal-distal physiological gradient of the endoneural fluid (47, 48). In fact, an axonal loss due to dying-back degeneration was observed and TTR aggregates are not steadily found in sural nerve biopsy despite the finding of axonal loss (50).

Schwann cells play a key role in early pathogenic changes in the peripheral nerves (49, 50). Some studies have shown that an atrophy of non-myelinating Schwann cells in direct contact with amyloid fibrils is evident in patients with early-onset ATTRv, suggesting that mechanical amyloid-induced damage to Schwann cells plays an important role in the loss of small-diameter nerve fibers (49). Differently, non-myelinating Schwann cell morphology is relatively well preserved in late-onset cases, even though amyloid deposits are in close contact with such cells (49, 51).

Since the Val30Met mutation leads to structural abnormalities in endothelial cells by inducing apoptosis and inhibiting their migration, a role for them, which are the first-line barrier to the circulating mutant transthyretin, has been hypothesized in starting large fiber damage characteristically in late-onset forms (47, 52).

## From clinical features to diagnostic approach

The clinical manifestations are heterogeneous and are influenced by several different factors, such as age of onset, genotype, penetrance of mutation, geographic origin, and disease duration.

In early-onset phenotypes, symptoms onset is between the 2nd and 5th decade of life while the late-onset phenotype shows clinical manifestation at >50 years of age and usually has variable penetrance, lack of family history, and fewer autonomic symptoms (17).

Potentially every organ can be affected by ATTRv with different severity, including peripheral nerves, the central nervous system, the heart, the eyes, the kidney, and the gastrointestinal system. In Nativi-Nicolau et al.'s study, it is underlined that patients with ATTRv have multiple symptoms and hospitalizations before diagnosis and a statistically significant difference was found comparing the comorbidities of ATTRv patients and matched controls in cardiovascular, musculoskeletal, gastrointestinal, metabolic, nervous system, and ocular systems (53).

**Figure 2:**
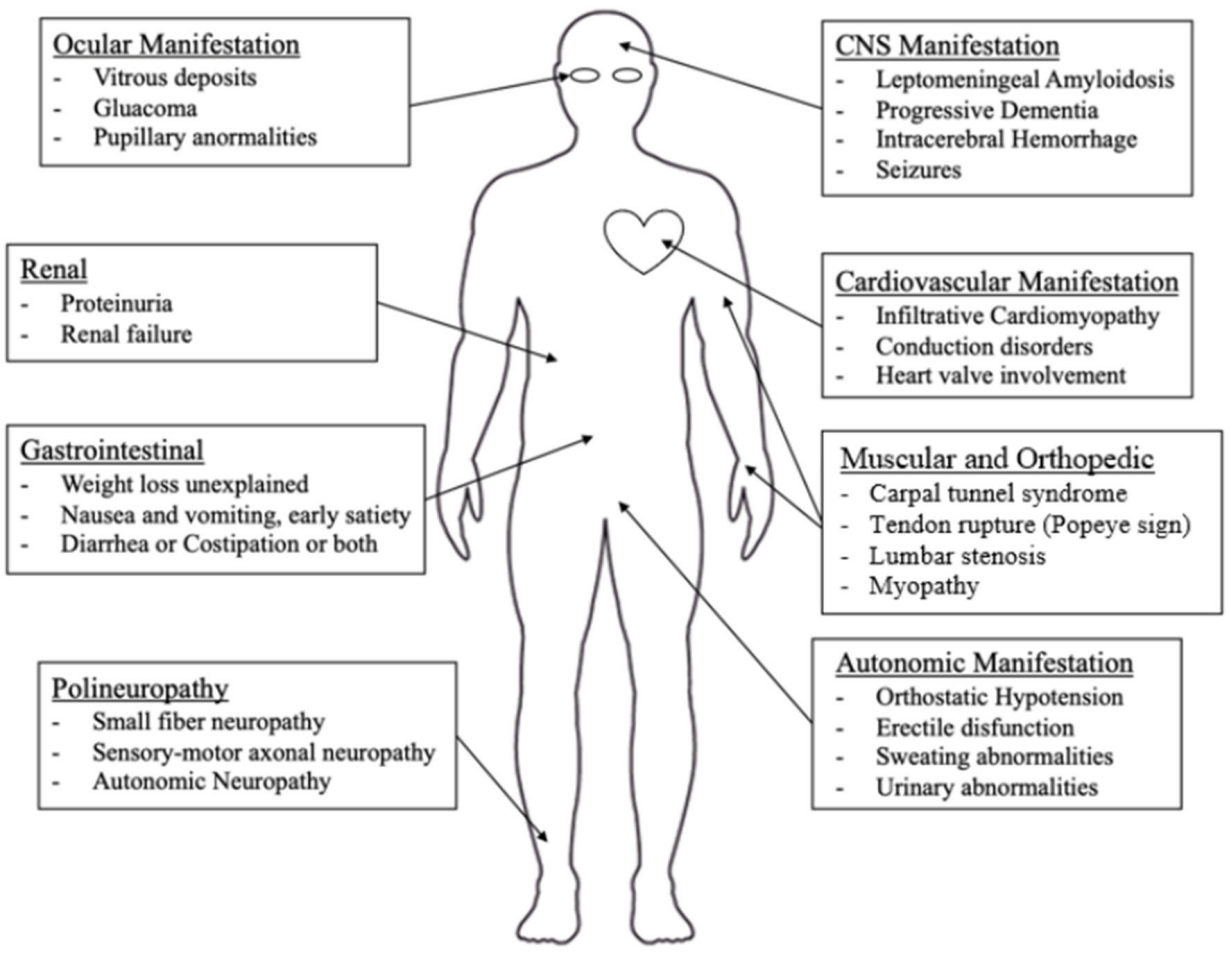
Main clinical manifestations of ATTRv.

Nonetheless, peripheral nerves and heart appear to be the main targets of the TTR-related pathological process (1, 3, 7, 53, 54). In Figure 2, an overview of the main clinical aspects of ATTRv is reported, which are detailed below.

### Neurological features of ATTRv

#### Small fiber neuropathy and large fiber polyneuropathy

Different neuropathic phenotypes in patients carrying the same mutation as Val30Met are reported in the literature (17, 54). In the early-onset phenotype, small fibers are first affected, which results in a typical small fiber neuropathy with autonomic involvement (17, 54). Following disease progression and damage to larger fibers, the involvement of all sensitivities and motor impairment become evident with length-dependent progression (17, 54, 55). Differently, in the Val30Met late-onset phenotype, larger myelinated fiber involvement is predominant over the small nerve fiber damage, thus prevalent symptoms are sensory loss in all modalities and early muscle atrophy and weakness, with consequently gait impairment (17, 54). Therefore, it has been hypothesized that late-onset and early-onset patients do not share the same pathomechanism (48).

Small-fiber neuropathy is characterized by neuropathic pain along with other positive sensory symptoms and impaired pain sensitivity, while tactile sensitivity and proprioception remain preserved (3, 7, 17, 56). The diagnosis for clinicians is guided by symptoms and neurological examination, eventually confirmed by skin biopsy (57).

Typically, the large fiber neuropathy associated with ATTRv is characterized by a rapidly progressive and disabling sensorimotor axonal neuropathy (1, 57, 58). Disease progression occurs faster than in other hereditary or idiopathic neuropathies (59). Severe neuropathic involvement increases disability as patients may finally be wheelchair-bound or bedridden (60).

#### Autonomic dysfunction

Autonomic involvement is related to early amyloid deposition in the DRG and sympathetic ganglia and is frequently described in the early stages of disease as the first clinical manifestation in some cases (49). Symptoms of autonomic involvement include orthostatic hypotension, reduced sweating, erectile dysfunction, dry eyes and mouth, bladder abnormalities, and gastrointestinal motility alterations (49, 61). Gastrointestinal symptoms are variable, as ATTRv patients may present with premature satiety, gastric distension, recurrent nausea and vomiting, diarrhea, and/or constipation (17, 49). In the late-onset forms, autonomic dysfunctions are often mild and may remain under-recognized if not properly investigated (49). Symptomatic treatments are required to reduce symptomatic disability and improve the quality of life of patients.

#### Carpal tunnel syndrome

Patients complaining of numbness and tingling at the hands along the median nerve distribution with or without evidence of muscle weakness at the right abductor pollicis brevis and positive Tinél and Phalen signs at neurological examination should raise the suspicion of carpal tunnel syndrome (CTS) (62). CTS is a very common compression neuropathy, affecting around 6% of the general population during their lives (63). ATTRv-related CTS is not simply caused by compression linked to amyloid fibrils (64, 65). Differently from idiopathic CTS, ATTRv patients showed a morpho-functional dissociation of the median nerve at ultrasound studies, as parameters of nerve enlargement did not correlate with neurophysiological severity (64, 65).

CTS has been recognized as an early, even if non-specific, manifestation of ATTRv (7, 62, 63). In the study by Karam et al. from 2019, CTS occurred in two-thirds of patients with ATTRv, and, more importantly, it may predate the diagnosis by up to ten years (63). Sperry et al. found amyloid deposits on tenosynovial tissue biopsy in 10 out of 98 patients (median age 68 years) undergoing carpal tunnel surgery for idiopathic carpal tunnel syndrome, and two of them were diagnosed with ATTRv (66).

Taken together, these findings support the concept that carpal tunnel syndrome should be considered a red flag for diagnosis, especially when it involves both sides, has an early onset without risk factors (such as hand-working activities or comorbidities), there is a positive family history, and/or is severe-extreme degree (1, 67).

#### Lumbar canal stenosis

Lumbar canal stenosis is the narrowing of the spinal canal, which leads to compression of nerves and eventually the spinal cord (68).

It usually affects people over 50 years old, with a prevalence of 1.7–13.1% in the general population (68, 69). The most frequent symptoms reported by patients are pain and weakness of the lower limbs, associated with cramping in the calves (68, 69).

Lumbar canal stenosis secondary to thickening of the flavum ligament may be an early clinical presentation of ATTRv (70–72).

As amyloid deposition potentially occurs in every tissue, including synovial tissue, it is not surprising to find amyloid deposition in patients undergoing orthopedic surgeries (73). In Yanagisawa et al. study on 95 flavum ligament specimens, all specimens resected from patients with spinal canal stenosis had amyloid depots, out of whom 45% were TTR positive (72).

Therefore, lumbar canal stenosis may be considered an element of suspicion of a possible accumulation of amyloid, even though it does not discriminate between genetically and non-genetically determined forms.

#### Myopathy

Although rarely encountered, myopathy is reported as a possible clinical manifestation of ATTRv as a result of muscle damage linked to amyloid accumulation in muscles, especially in the perimysium (74, 75). Affected patients develop proximal weakness and myogenic changes on electromyographic study (74, 75). Since it may not always be easy to distinguish clinically between muscle and nerve involvement, amyloid myopathy may be underdiagnosed (74, 75).

#### Central nervous system involvement

Although central nervous system (CNS) involvement is rare, leptomeningeal amyloidosis has been described mostly as associated with non-Val30Met mutations (Leu12Pro, Ala25Thr, Gly53Glu, Tyr114Cys, Asp18Gly, or Tyr69His) (72, 76, 77).

While before liver transplantation therapy, the average survival of patients was 7–10 years from disease onset, the increased survival secondary to transplantation allowed to unmask elective CNS manifestations that may become evident 15–20 years after the onset of systemic disease (78, 79). They are related to the continued production of amyloid by the retinal epithelium and choroid plexuses which is unaffected by transplantation (78, 79). TTR amyloid may deposit in the media and adventitia of the small cerebral arteries and veins, as well as at the subarachnoid and leptomeningeal levels (58, 80, 81). When findings of leptomeningeal amyloidosis associate with vitreous involvement, a syndrome called familial oculolectomeningeal amyloidosis (FOLMA) is defined (81).

Clinical manifestations are a consequence of the development of brain small vessel disease and may include ischemic stroke, cerebral and subarachnoid hemorrhage, cognitive impairment, ataxia, and epilepsy (58, 78, 79, 82). The most frequently reported manifestations are transient focal neurological episodes (TFNE, or “amyloid spells”), which are characterized by stereotyped, relapsing clinical episodes that tend to have negative symptoms (for instance sensory loss, focal motor weakness) (78, 79).

Magnetic resonance imaging (MRI) studies may detect leptomeningeal enhancement and eventually could be helpful to guide cerebral biopsy, while CSF analysis is not specific and usually shows moderate hyperproteinorrachia (83, 84).

It is important to keep in mind the possible cerebral involvement of ATTRv in elderly patients complaining of cognitive impairment or recurrent vascular events (82–84).

### Non-neurological features of ATTRv

#### Cardiac involvement

Severe cardiac involvement occurs more frequently in the late-onset phenotype and is associated with increased mortality risk (60). Typically, myocardial involvement is infiltrative due to amyloid accumulation (85, 86). The electrocardiogram in ATTRv patients may show an anterior “pseudo-infarction” pattern and T-wave abnormalities (85, 86). Conduction disorders such as branch block, atrioventricular block, and sinus-atrial block are also reported (85, 86). Amyloid accumulation leads to an increase in the thickness of the walls of the right and left ventricles and of the atrial septum (hypertrophic phenotype), which is detected by echocardiogram ultrasound (85, 86). Consequently, restrictive physiology puts patients at risk of diastolic heart failure as the disease progresses (85, 86).

Heart valve involvement is also common and often accompanied by valve regurgitation (85, 86).

Patients with the Val30Met mutation from endemic areas tend to have less severe cardiac involvement than patients with the same mutation from a non-endemic area or with no Val30Met mutations (85, 86).

Overall, cardiac involvement is very heterogeneous, affecting more severely late-onset phenotypes, and is one of the most important prognostic factors for survival in patients affected by ATTRv.

#### Ocular involvement

Among ATTRv patients, 10% present with ocular involvement that usually appears later in the course of the disease, such as vitreous opacities, chronic open-angle glaucoma (COAG), abnormal conjunctival vessels (ACVs), keratoconjunctivitis sicca (KCS) and corneal neuropathy (87).

A typical ocular pattern may be present in ATTRv called “scalloped pupil”, defined as irregular pupillary margins and fringed edges (88).

#### Renal involvement

Infiltrative renal amyloidosis causes nephrotic syndrome and progressive renal failure (10, 11, 89). Approximately one third of patients from endemic areas and 6% of patients from non-endemic areas present with renal involvement, which has been described only in the Val30Met ATTRv patients and is thought to be extremely rare in the ATTRwt (10, 11, 89).

Amyloid renal deposits were detected with different distributions including pericapsular, vessels, interstitium of the medulla and cortex, and tubular basement membrane (89).

#### Gastrointestinal involvement

The prevalence of gastrointestinal manifestations appears to be between 56 and 69%, depending on the type of mutation (82). Gastrointestinal manifestations are various, and the most common symptoms are diarrhea, steatorrhea, and involuntary weight loss, which often appear before developing symptoms related to neuropathy or constipation (12, 89). Less frequently, possible pseudo-obstruction (dysautonomic slowness of the gastrointestinal tract) has been described with a particularly severe prognosis, generally not responding to pro-motility agents (89). Hepatic involvement is frequent, usually presenting as a mild hepatomegaly and an elevated alkaline phosphatase level (89, 90).

### Diagnostic pearls and pitfalls

Clinical manifestations of ATTRv in endemic areas make diagnosis relatively easy in such a setting.

Not only are clinicians more likely to be aware of clinical characteristics because of the higher prevalence of ATTRv, but specific phenotypes are also well described in the literature.

In endemic areas, early-onset phenotypes are more frequent, and a positive family history is usually detected with high penetrance (7, 17, 44, 54). In these cases, the diagnosis is usually achieved within 1 year from the onset of the symptoms (7, 17, 44, 54). Differently, in non-endemic regions, diagnosis delay may be over 3 years longer for several reasons, such as negative family history, heterogeneity in presentation at onset, and more complex differential diagnosis in cases mimicking other forms of neuropathy (e.g., chronic inflammatory demyelinating polyneuropathy, CIDP) (44).

In this setting, the rate of ATTRv diagnosis within 6 months is lower, occurring in only 35% of patients, with more than a third of patients consulting more than five physicians before receiving a correct diagnosis (91).

### Neurophysiological features

The electrophysiological characteristics are variable because of different involvement patterns and may depend on the time at which the neurophysiological study is performed, making the diagnosis very difficult (58, 92). We suggest nerve conduction studies (NCS) should be performed in all four limbs, including studies of the median nerve bilaterally.

In the early-onset phenotype, the involvement of small nerve fibers is clinically relevant and the NCS may be normal, while in the late-onset phenotype an axonal damage of the large fibers is usually detected (54). Early involvement of the upper limb nerve, other than median nerve, seems more frequent compared with idiopathic polyneuropathy and could be regarded as a suspicion clue (93, 94). For these reasons, we suggest to always investigate an apparently idiopathic axonal polyneuropathy as a possible presentation of ATTRv.

A composite clinical and electrophysiological score to screen patients with ATTRv has been recently proposed. It is constituted by seven total items, ranging from 0 to 12. Authors set the cut-off ≥ 5 points to discriminate ATTRv patients with a sensitivity of 96.6% and a specificity of 63.6% (94).

Although axonal damage is the most reported feature, demyelination has also been described and may lead to misdiagnosis with other acquired or hereditary polyneuropathies (95).

### Imaging of the peripheral nerve

#### Ultrasound study

Through the ultrasound study of the median nerve, a distinctive CTS pattern has been described in ATTRv with respect to the idiopathic form (64). In ATTRv-linked CTS, an inverse correlation between cross-sectional area and severity of CTS was observed, which may be related to ischemic nerve damage after amyloid deposition (64, 96). Most of the nerves have been found to be significantly larger than those of carriers, mainly at proximal sites both in the upper and lower limbs (64). Although polyneuropathy is a distal length-dependent process, this finding may be explained by the focal pattern of amyloid deposits that are located at proximal sites (64).

#### MRI neurography

MRI neurography (MRN) is a promising method to identify lesions of the peripheral nerve resulting from the accumulation of amyloid (97). The first studies of the MRI approach in ATTRv started in the early 2010s, detecting focal hyperintensity in the proximal nerves of four limbs compared to healthy controls (97, 98). In the Kollmer et al. study, signal quantification at proximal sites (thigh level) detected a significant increase in proton density and T2-relaxation time in symptomatic patients, while asymptomatic carriers showed only increase in proton density (97). Recently, ATTRv-PN was differentiated from ATTRv pre-symptomatic carriers and healthy controls by using MRN and DTI of the sciatic nerve (98). The method was also able to detect subclinical changes in asymptomatic carriers, thereby facilitating early diagnosis and evaluation of progression (99).

### Imaging of the heart

#### Ultrasound study

Ultrasonography is certainly the first-line, less expensive, and most accessible method of approaching a patient with suspected cardiac amyloidosis (100, 101). The main cardiac structural findings secondary to amyloid accumulation, although nonspecific, include granular/scintillating appearance of the left ventricle (LV), bilateral atrial enlargement, and ventricular wall thickening, particularly at the level of the septum, with preserved ejection fraction, at least in the early stages of disease (100, 101). The measurement of ventricular myocardial stiffness, known as longitudinal strain, seems to be very useful in differential diagnosis since, in patients with amyloidosis, there is an increase in cardiac muscle stiffness secondary to amyloid accumulation (100–103). “Apical sparing” is considered a pattern highly suggestive of amyloidosis, with greater involvement at the base and in the mid-ventricular regions than at the apex, and may differentiate amyloid cardiomyopathy from other forms of hypertrophic heart disease (102, 103).

#### Cardiac magnetic resonance imaging

Cardiac magnetic resonance (CMR) is an advanced method for studying the structure and function of the heart and is useful in confirming the changes already visible on the ultrasound studies, such as left ventricular hypertrophy, atrial dilatation, and diastolic dysfunction (101, 102). After gadolinium-based contrast medium administration, a rather characteristic pattern was reported with initial subendocardial late gadolinium enhancement (LGE) followed by transmural enhancement (104). LGE allows a diagnosis of cardiac amyloidosis with high sensitivity (85%) and specificity (92%) (105). However, some limitations in performing CMR may include moderate/severe renal failure, poor patient compliance, and incompatible intracardiac devices (102–105).

#### Bone scintigraphy

Bone scintigraphy using technetium-labeled bisphosphonates (99mTc-labeled 3,3-diphosphono-1,2-propanodicarboxylic acid (DPD), 99mTc-labeled pyrophosphate (PYP), and 99mTc-labeled hydroxymethylene diphosphonate (HMDP)), is a well-established tool to detect cardiac amyloidosis (106–111). After incidentally finding uptake of bone tracer in a patient affected by familial amyloidotic polyneuropathy, a small group of ATTRv patients underwent bone scintigraphy in 2002, showing increased extra-osseous uptake in the heart, hips, and shoulder (106). A recent study reviewed bone scintigraphy scans performed for any clinical indication from 2009 to 2020, unveiling cardiac uptake in 0.7% (107). Myocardial uptake has been shown to be sensitive in the early phases of ATTRv and it is calculated by a semi-quantitative method with a visual comparison between cardiac and bone uptake and stratified into grades according to Perugini's classification (106–111). A higher grade corresponds to a higher uptake (109). The finding of increased cardiac uptake on scintigraphy has >99% sensitivity in diagnosing ATTR, but with a low specificity (about 68%) compared with endomyocardial biopsy (109). Although positivity was also found in patients with other forms of amyloidosis, especially AL amyloidosis, in the latter condition the uptake is typically low grade; thus, as the degree of uptake increases, the specificity of the test for the diagnosis of ATTR increases (110).

Although positivity of bone scintigraphy with a medium to high degree uptake, in the absence of monoclonal components in serum and urine, is considered highly suggestive for ATTRv, in some specific mutations bone scintigraphy may have low sensitivity, even in the presence of cardiac involvement, i.e., the Phe64Leu variants (110, 111). The mechanism of this low avidity of the tracer for individual mutations is not entirely clear (111).

### Biopsies

Amyloid deposits are detected by Congo Red staining in different tissues, mainly fat aspirate and salivary gland tissue but also nerve, skin, myocardium, kidneys or gastrointestinal mucosa (112). On tissue confirmation of TTR amyloidosis is useful in cases of concomitant monoclonal serum or urine components and can be obtained by immunohistochemistry or mass spectroscopy in order to differentiate ATTRv from other forms of hereditary or acquired amyloidoses (112). However, false negative or false positive results may be observed due to the irregular distribution of amyloid in the tissues (112).

Fat aspirate are widely used for the diagnosis of systemic amyloidosis. It has the advantage of being a simple and low-cost procedure with high specificity, but its sensitivity is variable (113). In Quarta et al. study on 600 patients affected by cardiac amyloidosis, fat aspirate tested positive in < 45% of ATTRv patients and about 15% of ATTRwt subjects (113).

A biopsy can be performed on a minor salivary gland having a sensitivity of about 90% (25, 114). In a large clinicopathological study, TTR amyloid deposits were found less frequently than other systemic amyloidoses, yet interestingly, non-V30M mutant ATTRv and ATTRwt were strongly associated with amyloid nodules located in close contact with salivary excretory ducts (114).

Nerve biopsies are usually performed on sural nerves and may reveal primary axonal neuropathy with possible secondary demyelination, axonal regeneration, and remyelination, together with endoneurial amyloid deposits and vascular alterations with a sensitivity of about 80% (25, 114, 115).

Skin is a target tissue for minimal invasive biopsies that have a sensitivity of about 70% and may detect amyloid as a marker of disease onset (25, 112).

Taking into account the varying sensitivity of biopsy techniques, the diagnosis should not rely on biopsy findings when the clinical suspicion is strong and supported by other surrogate tests (e.g., bone scintigraphy).

### Genetics

The molecular test for the *TTR* gene on blood samples is now the gold standard to minimize lost time (1). The detection of known pathogenic mutations virtually leads to a conclusive diagnosis (1). Otherwise, any finding of variants that are not known or of undefined clinical significance can be further investigated with histochemical studies on tissue. As ATTRv is a severe but treatable disease, testing of potential pre-symptomatic carriers is suggested (116).

### Diagnostic approach and differential diagnosis

The differential diagnosis of typical length-dependent axonal polyneuropathy includes many acquired conditions such as diabetic, vasculitic or uremia neuropathies as well as hereditary neuropathies such as axonal Charcot-Marie-Tooth disease (48). Familial and patient history has to be collected, paying attention to ATTRv “red flags” (e.g., dysautonomia, bilateral tunnel carpal syndrome).

A rapid progressive course represents a relevant clinical aspect for raising the suspicion of ATTRv, especially in late-onset phenotypes that, once clinically manifested, worsens faster than the early-onset phenotype and the other peripheral neuropathies considered for the differential diagnosis (117, 118).

**Figure 3:**
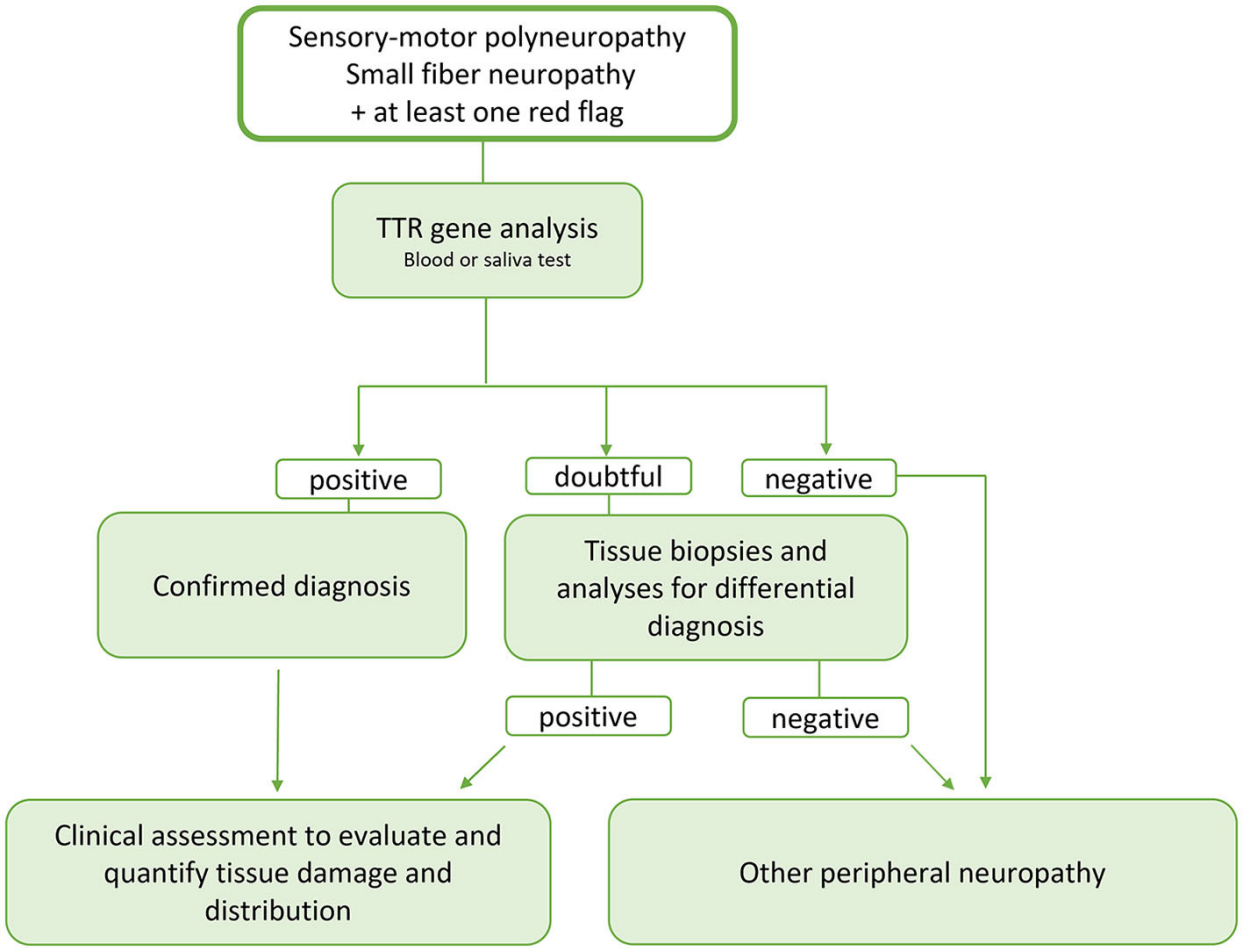
Schematic flow-chart for the neurologist's diagnostic approach to ATTR. Since ATTR is a potentially treatable disease and the therapy is more effective the earlier it is started, we propose an early genetic test, now easily and rapidly available, in the presence of signs of involvement of the peripheral nervous system and at least one red flags. In case of doubtful response, the diagnostic tools available today such as bone tracer scintigraphy, evaluation of a possible multisystem involvement and tissue biopsy studies can help in better defining the diagnosis and evaluating a possible tissue accumulation of TTRwt. At the same time and, in case of negativity of the genetic study or aforementioned tests, analyses must be started to identify possible other causes of peripheral neuropathy including the search for monoclonal gammopathy.

Main neurological, cardiological and general “red flags” are listed in Table 1 and a proposal of schematic flow-chart for the neurologcal diagnostic approach is reported in Figure 3.

**Table 1:** Main red flags in diagnosing ATTRv.

Multisystem involvement red flags	Unexplained weight loss (>5 kg)
	Gastrointestinal motility disorders
	Vitreous deposits
	Kidney disorders (proteinuria, kidney failure)
Neurological red flags	Family history of peripheral nerve disease (especially if associated with cardiac involvement)
	Neuropathic pain and/or progressive sensory disturbances
	Autonomic dysfunctions (erectile dysfunction, orthostatic hypotension, neurogenic bladder)
	Idiopathic bilateral carpal tunnel syndrome
	Biceps tendon detachment and/or trigger finger
	No response to immunotherapy (when the clinical suspicion is CIDP or other immune-mediated polyneuropathy)
	Rapid disease progression
Cardiological red flags	Positive family history
	Cardiac hypertrophy; left ventricular wall thickness ≥12mm
	Arrhythmia
	Hypotension or normotension if previously hypertensive; lipothymias
	Paradoxical low flow/low gradient aortic stenosis
	Excessively elevated NT-proBNP relative to the degree of heart failure
	Persistent elevated troponin levels
	Discordance between EKG voltage and wall thickness
	Atrioventricular conduction disorder
	Intolerance to ACE inhibitors and beta blockers

Laboratory tests should always be performed to rule out acquired neuropathies and elements suggestive for amyloid deposition must be systematically searched both in the neurological and in cardiological and gastroenterological setting.

Diagnosis may be challenging if other hematological disorders are suspected (e.g., POEMS, AL amyloidosis) or if genetic testing is not conclusive (detection of novel variants or VUS), so second- or third-level investigation such as MRI neurography or target tissue biopsy may be needed (48, 119).

In cases of prevalent demyelinating changes at electroneurography, ATTRv neuropathy may be misdiagnosed for CIDP (48, 119). A large cohort study compared ATTRv clinical manifestations to CIDP and POEMS ones (120). Neuropathic pain was far more frequently reported and more severe in ATTRv patients than in CIDP patients, but severe pain was also observed in POEMS syndrome (120). The presence of dysautonomia appears to be more distinctive for differentiating ATTRv from CIDP/POEMS (120). In nerve conduction studies, reduced sensory conduction velocity of the median and ulnar nerves was more frequent in ATTRv (120). In Du et al. study, the pattern of myelinated nerve fiber density (MFD) in sural nerve biopsies of 41 ATTRv patients was compared to other neuropathies (95). MFD was milder in CIDP than ATTRv, with a slower MFD reduction per year (95). Overall, differentiating ATTRv from CIDP may be challenging, but severe neuropathic pain and dysautonomia should raise suspicion for ATTRv.

## From treatment to follow-up

Symptomatic therapy and disease-modifying treatments aimed at preventing further production of amyloid should be considered after every diagnosis.

A few disease-modifying therapies have been developed over the past 30 years.

Liver transplantation was first applied to suppress the production of mutant TTR in 1990 (121).

Subsequently, stabilizing drugs of the tetrameric TTR structure were developed, such as Diflunisal and Tafamidis. The first is a non-steroidal anti-inflammatory drug, and the latter is a small molecule designed to stabilize TTR (122, 123). They share a similar mechanism to stabilize TTR by occupying thyroxine-binding sites of circulating TTR proteins (122, 123).

More recently, *TTR* gene silencers have been released aimed at blocking mRNA synthesis and reducing the production of TTR (124).

### Liver transplantation

The first report on the outcome of liver transplanted ATTRv patients was in 1993 (125). Since then, hundreds of transplants have been performed, mostly in patients with Val30Met mutation (121). Long-term transplantation outcomes included in FAP World Transplant Registry showed that survival at 20 years was 53.3%, certainly higher than the natural history of disease and that independent predictive factors for survival were lower age at transplantation, short disease duration from onset, higher mBMI and presence of Val30Met mutation (121). The main causes of mortality were septicemia (22%) and cardiovascular (22%) and transplant-related complications (14%) (121). Although survival in transplanted patients was prolonged when performed in the early stages, limited efficacy in reducing neuropathy and cardiopathy progression has been observed that continued after liver transplant (121, 126).

Finally, CNS and eye damage also tended to progress or appeared after transplantation, resulting from local TTR production unaffected by liver transplantation (78, 79).

### TTR stabilizers

#### Diflunisal

Diflunisal is a nonsteroidal anti-inflammatory, salicylic acid derivative, which acts by inhibiting cyclooxygenases 1 and 2 thereby mediating the inflammatory response (127). It was evaluated at a dose of 250 mg twice daily in a phase 1 study, proving effective in stabilizing the TTR protein in its native state, thereby reducing its degradation (127).

Later, Diflunisal was administered in a 2-year, randomized, double-blind, placebo-controlled trial in 130 patients with ATTRv (64 Diflunisal vs. 66 Placebo) (128). The study showed a statistically significant reduction in neuropathy progression, as assessed by using specific scales (Neuropathy Impairment Score plus 7, NIS+7) (128). Currently, the drug is not approved for use in patients with ATTRv, so it can only be used off-label. Chronic use of Diflunisal is somewhat limited because of inpossible gastrointestinal and renal adverse effects (129).

#### Tafamidis

Tafamidis is a molecule that can selectively bind TTR in plasma, making it more stable and preventing dissociation into monomers and thus amyloidogenesis (130, 131).

The efficacy of Tafamidis in TTRv was tested in an 18-month randomized, double-blind, placebo-controlled trial (123). A total of 128 patients were enrolled (65 Tafamidis vs. 63 Placebo), all with Val30Met mutation with early onset phenotype, signs of peripheral polyneuropathy and fair overall clinical status (Karnofsky performance status ≥50) (123). When administered at a dose of 20 mg once daily, no significant difference in the primary outcome, probably due to the high number of dropouts and the short observation period was observed (123). However, improvement in small and large nerve fiber function in individuals with TTR-FAP, slowing the disease's course and lessening the severity of the condition (123).

Following these results, other studies were conducted in patients with mutations other than Val30Met and with late-onset phenotype showing slowed disease progression even if not arrested (132–135). In all the studies, the side effects were superimposed on the placebo group, with no serious adverse events even in the long-term follow-up (132–135). Based on these results, Tafamidis has been approved in Europe and some other regions of the world for early forms of ATTRv.

In 2018, Tafamidis was tested in a ATTR-ACT trial demonstrating a significant reduction in mortality and hospitalization related to cardiological issues, in association with lower disease progression and reduced decline in quality of life (136).

For these reasons, it was approved by FDA and subsequently by EMA for ATTRv-CM.

### Molecular therapy

Gene silencing strategies developed to treat ATTRv are focused on the use of antisense oligonucleotides (ASO) or small interfering RNA (siRNA) (124). Two drugs, Inotersen (ASO) and Patisiran (siRNA), have been recently approved for the treatment of mild and moderate stages of ATTRv (124).

#### ASO

Inotersen is an ASO that has the ultimate effect of reducing circulating TTR levels, resulting in the limitation of tissue deposition (130–132). It selectively binds TTR mRNA causing its degradation and thereby inhibiting synthesis of both mutated and wild-type form, and is administered weekly subcutaneously (130–132). It has been approved by the FDA and EMA for treating ATTRv-PN after the promising results of the NEURO study -ATTR (137). This randomized controlled trial enrolled 172 patients and showed statistically significant differences in the modified Neuropathy Impairment Score+7 (NIS +7) and quality of life scales between the Inotersen group and the placebo group after 15 months of follow up (137). Specifically, in the Inotersen group, 36.6% of patients had a stable or improved NIS+7 compared with the 19.2% of patients in the placebo group (137). Moreover, in patients with moderate to severe cardiomyopathy, no signs of cardiac disease progression emerged (54, 56, 57).

Three patients in the Inotersen arm developed glomerulonephritis, while one patient developed a fatal cerebral hemorrhage associated with reduced platelet count (<10,000 per cubic millimeter) (137). These findings should prompt to check and monitor blood count and renal function before starting the drug and during treatment. However, the real-life data are encouraging in that they confirm the negative effect on platelets but in the absence of serious adverse events (138–140).

#### siRNA

Patisiran is a siRNA that specifically targets a genetically conserved sequence in the 3′ untranslated region of all variant and wild-type TTR mRNA (141). Patisiran is formulated as lipid nanoparticles and is administered via intravenous infusion and transported by blood to the liver, the primary source of TTR protein in the circulation (141). The apolipoprotein opsonized in the lipid capsule of the nanoparticle is recognized by the apolipoprotein-E receptors of hepatocytes, and the drug is internalized by endocytosis. that delivers the siRNA to hepatocytes (141). Through the natural RNA interference (RNAi) process, Patisiran causes the catalytic degradation of TTR mRNA in the liver, resulting in a reduction of serum TTR protein (141). Patisiran was first approved for therapeutic usage by the FDA and the EMA in August 2018, for the treatment of mild or moderate (stages I and II) ATTRv-PN (58, 142).

In the phase III APOLLO placebo-controlled study, the efficacy of Patisiran (0.3 mg/kg every 3 weeks for 18 months) was tested, and a total of 225 patients were randomly assigned to receive Patisiran or placebo, accounting for clinical severity and genotype (142). Improvement from baseline in mNIS + 7 scores occurred in 56% of participants in the Patisiran group and only 4% of those who received placebo, along with improvement in Norfolk QoL scores (51.4% of patients in the Patisiran group vs. 10.4% in the placebo group), in gait speed (53% of patients who received Patisiran vs. 13% of those who received placebo), and in the COMPASS-31 measure of autonomic symptoms (143). In a 126-patient subpopulation with cardiac involvement, Patisiran reduced the thickness of the left ventricular wall by 0.9 ± 0.4 mm, reduced overall longitudinal deformation by −1.4 ± 0.6%, and increased cardiac output by 0.38 ± 0.19 L/min (144).

An ongoing trial involving Patisiran is the APOLLO-B study (NCT03997383), a multicenter, phase III, randomized, and placebo-controlled study that investigates the drug in ATTR-CM patients, with an estimated study completion date of June 2025 (58).

Common adverse reactions occurring with Patisiran include mild to moderate peripheral edema and infusion-related reactions.

Vutrisiran blocks the production of TTR by targeting and silencing specific messenger RNAs (145). An open-label multicenter study (HELIOS-A) compared the efficacy of vutrisiran administered by subcutaneous injection once every 3 months to 122 patients and Patisiran (0.3 mg/kg) by intravenous infusion once every 3 weeks for 18 months to 42 patients (145).

The treatment with Vutrisiran met the primary endpoint (improvement in the modified mNIS+7 at 9 months) and achieved statistically significant results on secondary measures such as the Norfolk Quality of Life Questionnaire-Diabetic Neuropathy (Norfolk QoL-DN) and the timed 10-meter walk test (10-MWT) improvement (145).

The most reported adverse events included arthralgia, dyspnea, and a decrease in vitamin A; mild and transient reactions at the injection site were reported in five patients (145).

The drug developer announced that HELIOS-A met all secondary endpoints measured at 18 months, thus the FDA approved the drug for ATTRv-PN in June 2022 and EMA in September 2022.

### Upcoming therapeutic options

#### Eplotersen

Eplotersen is a ligand-conjugated ASO that can inhibit transthyretin production. It is administered subcutaneously every 4 weeks (124). In the NEURO-TTRansform Study, the drug showed statistically significant results in both reducing TTR values and blocking clinical worsening (assessed by NIS+7) with improvement in quality of life, in 144 patients treated for 66 weeks (146). The safety profile was superimposable to that of placebo (146).

#### CRISPR–Cas9 editing

The CRISPR/Cas9 system is based on the use of the Cas9 protein, which can break DNA and can be programmed to make specific changes to the cell genome (124). The molecule is introduced into the cell through an RNA molecule that acts as a guide (guide RNA) (124).

NTLA-2001, an *in vivo* gene-editing therapeutic drug based on the Clustered Regularly Interspaced Short Palindromic Repeats and Associated Cas9 Endonuclease (CRISPR-Cas9) system, consists of a single guide RNA targeting TTR and a lipid nanoparticle encasing messenger RNA for the Cas9 protein (147). Targeted knock-out of TTR caused serum TTR protein concentrations to drop in a small sample of individuals with hereditary ATTR amyloidosis with polyneuropathy with very moderate side effects (147).

#### AG10

AG10 (Acoramidis) is a highly selective orally taken TTR stabilizer that has been created to mimic the hyperstabilizing protectiveeffect of the T119M variant (148, 149). A randomized, double-blind, placebo-controlled study evaluating safety and tolerability of AG10 in ATTRv-CM patients with symptomatic chronic heart failure showed that the treatment was well-tolerated and achieved near-complete TTR stabilization especially at the higher doses (149). Consistently positive results from a phase 3 ATTRibute-CM study for patients with transthyretin amyloid cardiomyopathy (ATTR-CM) were recently announced and results should be submitted to the US FDA before the end of 2023 (150).

### Management and outcome measures

To monitor the disease evolution in clinical practice and to define the efficacy of treatment in a “real-world” setting, robust outcome measures are needed (151).

ATTRv management requires a multidisciplinary approach, including regular neurological, cardiac, renal, and ocular evaluations. A comprehensive list of assessments for monitoring progression in somatic and autonomic neuropathy and cardiac dysfunction (including the related indicators of progression, sensitivity to progression, suggested frequency of assessment and clinically significant worsening that may prompt a change in therapy) has been recently described and includes different neurological scales (R-ODS Rasch-built Overall Disability Scale; PND polyneuropathy disability; SFN-SIQ small-fiber neuropathy and symptom inventory questionnaire; NIS neuropathy impairment score; Sudoscan; COMPASS-31 Composite Autonomic Symptom Score-31; 10MWT and 6MWT 10/6-min walking test) as well as cardiac tools (12-lead ECG, cMRI cardiac magnetic resonance imaging, KCCQ Kansas City Cardiac Questionnaire) and other general health measures (eGFR estimated glomerular filtration rate, mBMI modified body mass index, Norfolk QOL-DN Norfolk Quality of Life-Diabetic Neuropathy, SF-36 36-item Short-Form Healthy Survey) (151).

The FAP staging system is widely used for hATTR and focus on the progressive walking impairment and neuropathy severity throughout the disease (32, 63). It was developed in Portugal in 1980 and considers three stages: (1) FAP 1 is defined by unassisted walking with mild bilateral foot and leg neuropathy; (2) FAP 2 is defined by the need for assistance in walking by crutches or canes with neuropathy spreading throughout the body; (3) FAP 3 is the most severe stage in which the patient becomes wheelchair-bound or bedridden and the neuropathy is severe. Although FAP is widely used, it is not sensitive enough to monitor short-term progression (43, 53, 152).

An interesting score system is the Neuropathy Impairment Score (NIS) which was designed to provide standard, quantitative, and overall scores of neurological impairments for continuous patient assessment, predominantly in clinical trials and epidemiological studies (63). NIS includes weakness, reflexes, and sensory evaluation as clinical variables, with a greatest emphasis on weakness. Total NIS goes from a minimum score of 0 to a maximum score of 244, indicating greater impairment.

The NIS-lower limb (NIS-LL) score is a subset of the NIS that evaluates the same variables only in the lower limbs (63, 153).

To overcome some NIS and NIS-LL limitations, NIS+7 was created. It considers the same NIS items added to seven other assessments that have been included to better characterize and quantify the neuropathic damage (63). Five of these measures are focused on three lower limb nerves: distal motor latency of the tibial nerve; amplitude of the compound action potential of the peroneal nerve; distal motor latency and conduction velocity, and amplitude of the action potential of the sural sensory nerve. The other two additional components of NIS+7 are the vibration detection threshold (VDT), a sensory measurement taken at the big toe, and the heart rate response to deep breathing (HRdb) as a measurement of autonomic involvement (63).

Modified NIS+7 (mNIS+7) Alnylam and mNIS+7 Ionis were created specifically for ATTRv randomized controlled trials. They were used as primary endpoints in two Phase III ATTRv-PN studies. mNIS+7 considers the same ratings and scores as the NIS+7 but discards the VDT for evaluating sensation and includes smart somatotopic quantitative sensation testing (SSTQST) (43, 62).

A current hot topic is the optimal timing for starting therapy in pre-symptomatic subjects with pathogenic variants in *TTR* and positive family history who are at risk of developing symptoms in the future. A consensus approach suggested to offer genetic testing in the context of genetic counseling to pre-symptomatic relatives aged 18 or older and recommended a regular follow-up not later than 10 years before the PADO for positive subjects which has to include baseline visit followed by a second visit after 6 months if symptoms are not reported, then every 12–24 months, and more frequent (6–12 months) as the predicted age of onset of symptomatic disease approaches (154).

## Conclusions

ATTRv is a severe systemic disease, primarily affecting the peripheral nerves and the heart, distributed throughout the world, and clinically and genetically heterogeneous.

Heterogeneity makes the disease difficult to diagnose, often resulting in enormous loss of time and increased disability for the patient.

Advances in molecular genetics have led to the discovery of molecules that have been shown to be effective in controlling the disease and improving several clinical aspects. Most patients benefit from treatment regardless of the stage of the disease, but early therapy is recommended to minimize organ damage. Therefore, a timely and definite diagnosis is essential in view of the availability of effective therapies that have revolutionized the management of affected patients.

Therefore, the goal of clinicians must be to intercept the signs of the disease early, suspect and diagnose it as early as possible in order to start treatment as soon as possible.

Future challenges will be evaluating effective therapy for patients in advanced stages of the disease, determining the impact of gene therapy, and assessing the potential benefits of combined therapies using drugs with different mechanisms of action, as well as the effects of disease-modifying therapies in pre-symptomatic individuals.

## Author contributions

LP, MF, and AP contributed to conception and design of the study. LP and BL wrote the first draft of the manuscript. SCP, FC, BR, and SD performed bibliographic research and wrote sections of the manuscript. MF and AP contributed to revision of the draft. All authors contributed to manuscript revision, read, and approved the submitted version.
